# Impact of Perioperative Multiple Doses of Glucocorticoids on Peripheral Blood Lymphocyte Subsets and Inflammatory Cytokines in Patients With Non-small Cell Lung Cancer

**DOI:** 10.3389/fsurg.2022.859984

**Published:** 2022-03-24

**Authors:** Liuquan Yang, Yixin Cai, Xiangning Fu

**Affiliations:** Department of Thoracic Surgery, Tongji Hospital, Tongji Medical College, Huazhong University of Science and Technology, Wuhan, China

**Keywords:** non-small cell lung cancer, glucocorticoids, lymphocyte subsets, inflammatory cytokines, lung resection

## Abstract

**Purpose:**

Surgery-induced immunosuppression is associated with infectious complications and cancer recurrence. This study aimed to characterize the effects of perioperative multiple doses of glucocorticoids on the peripheral immune environment in patients with non-small cell lung cancer.

**Methods:**

In this retrospective study, surgical patients with lung cancer were included. Lymphocyte subsets, lymphocyte phenotypes, lymphocyte functions, and inflammatory cytokines were evaluated in the peripheral blood preoperatively, then at 1 day and 7 days postoperatively. Levels of immune cells and inflammatory factors were compared between those who did or did not receive glucocorticoids at all time points.

**Results:**

Multiple doses or high doses (15–20 mg dexamethasone equivalents) of glucocorticoids that were all given within 24 h were associated with decreased absolute numbers of T cells, CD4^+^and CD8^+^T cells, B cells, and impaired T cells function at 1 day postoperatively while a single intraoperative low dose (5 mg) of dexamethasone had little influence on the peripheral environment. IL-1β, IL-6, and TNF-α were also more affected by multiple doses of glucocorticoids.

**Conclusions:**

Among patients with lung cancer, perioperative multiple doses of glucocorticoids that are all given within a short time are associated with decreased immune cell counts and impaired T cells functions.

## Introduction

Although medical treatment of non-small cell lung cancer (NSCLC) is rapidly developing with an increasing role for immune and molecular target therapies, surgery remains a mainstay in the cure and control of operable lung cancer. However, there is still a high risk for disease recurrence following surgery even in patients with early-stage NSCLC (1), the outcomes are poor and the mechanism of tumor recurrence remains unclear.

Dynamic changes of peripheral lymphocytes and cytokines in response to tumor removal have been noted following a variety of surgeries (2, 3). Such immune alterations not only lead to increased susceptibility to infection but also seem to provide fertile ground for both capture of residual malignant cells and their subsequent growth (4). Thus, the environment generated after cancer surgery could affect long-term cancer-related outcomes (5). However, perioperative factors associated with peripheral immunologic derangements have not been well-described, with the majority of investigations focusing on the impact of the surgical approach.

Glucocorticoids (GCs) are effective anti-inflammatory drugs widely used in surgical patients to treat a variety of indications (6–8), including prevention of postoperative nausea and vomiting (9), dyspnea, allergies, and edema. Hence the total times and dose of GCs may accumulate in a short time during the perioperative period. Given the immunosuppressive properties of GCs (10), there is understandable concern that the multiple doses of GCs may deepen already existing injuries by surgical trauma (11) and cancer (12) in the immune system of patients with NSCLC. Studies have reported that a single dose of GCs had few adverse effects on the short-term or long-term outcomes (13–15). Yet there are no data to date that evaluate how multiple doses GCs, especially all given within a short time, affect the surgery-induced immunosuppression in oncological patients. Therefore, we aimed to assess the effect of perioperative administration of multiple doses of GCs on the immune response to surgery in patients with NSCLC by measuring lymphocyte subsets, lymphocyte phenotypes, lymphocyte functions, and inflammatory cytokines preoperatively and at 1 day, 7 days postoperatively.

## Patients and Methods

### Patients

Patients with clinical stage IA-IIIA NSCLC who underwent minimally invasive lung resection from December 2020 to May 2021 at Tongji Hospital were evaluated. Clinicopathologic characteristics, including gender, age, smoking history, histology, tumor stage, epidermal growth factor receptor (EGFR) mutation status, and extent of resection were collected for all patients. Lymphocyte subsets, lymphocyte phenotypes, lymphocyte functions, and inflammatory cytokines were prospectively evaluated in peripheral blood at 6:00 a.m. on the surgery day, then at 6:00 am.. on the first day and seventh day postoperatively. Postoperative complications in patients with NSCLC have been previously described (16) and pulmonary, cardiovascular, and infectious complications were included in the present study.

Records of 152 patients were reviewed to determine if patients had received any oral or intravenous GCs between the first time point and the second time point of blood sample collection. Forty-four patients who were treated with GCs between the second time point and the third time point were excluded considering the comparability between the groups at all time points. Three patients who were receiving systemic GCs treatment for other immune diseases were also excluded. Hundred and five patients who met the inclusion criteria were then divided into GCs group (67 patients) and no GCs group (38 patients) based on the GCs administration. Information about the type and dose of GCs, repeated times of GCs, route of administration, and indications were collected.

### Ethics Approval

This study received ethical approval from the Medical Ethics Committee of Tongji Hospital, Tongji Medical College, Huazhong University of Science and Technology (TJ-IRB20210837).

### Lymphocyte Subset Analysis

The percentages and absolute numbers of total lymphocytes, T cells, CD4^+^T cells, CD8^+^T cells, B cells, and NK cells were obtained as follows: (1) 50 μL of whole blood was labeled with antibodies (anti-CD45, anti-CD3, anti-CD19, anti-CD16CD56, anti-CD4, anti-CD8) for 15 min at room temperature. (2) 450 μL of BD FACS lysing solution was added to the tube. (3) samples were analyzed using BD FACSCanto flow cytometer.

### Lymphocyte Phenotype Analysis

For each patient, two blood samples were processed and analyzed for different lymphocyte phenotypes. (1) 100 μL of whole blood was labeled with antibodies, cells in tube 1 were labeled with anti-CD45, anti-CD3, anti-CD4, anti-CD25, anti-CD127, anti-CD45R0, anti-CD45RA, cells in tube two were labeled with anti-CD45, anti-CD3, anti-CD4, anti-CD8, anti-CD28, anti-HLA-DR. (2) Incubated for 20 min at room temperature, the cells were then added with BD FACS lysing solution. (3) after being washed and re-suspended in 200 μL of PBS, the cells were analyzed using BD FACSCanto flow cytometer.

### Lymphocyte Function Analysis

IFN-γ production has been demonstrated to be well-correlated with various activities of lymphocytes and therefore lymphocyte function can be detected based on IFN-γ secretion (17). The percentages of CD4^+^IFN-γ^+^ T cells and CD8^+^IFN-γ^+^ T cells among total T cells, the percentages of NK IFN-γ^+^cells among total NK cells were obtained as followed: (1) the cells were stimulated for 4 h with a polyclonal cell activation mixture (Leukocyte Activation Cocktail, with BD GolgiPlug™) (2) the cells were stained with anti-CD45 anti-CD3, anti-CD4, anti-CD8, anti-CD56 (3) the cells were fixed and permeabilized (4) the cells were stained with intracellular anti-IFN-γ (5) the cells were analyzed with BD FACSCanto flow cytometer. The absolute numbers of CD4^+^IFN-γ^+^T cells were calculated by multiplying the percentages by the total CD4^+^T cell count, and the absolute number of CD8^+^IFN-γ^+^T cells were calculated by multiplying the percentages by the total CD8^+^T cell count. The absolute numbers of NK IFN-γ^+^cells were calculated by multiplying the percentages by the total NK count.

### Inflammatory Factors Analysis

Blood samples were processed and analyzed using the Siemens IMMULITE 1,000 Immunoassay system. Analyzed cytokines included IL-1β, IL-2R, IL-8, IL-10, and TNF-α. Levels of IL-6 were measured using the electrochemiluminescence immunoassay method (Cobas E602, Roche).

### Statistical Analysis

After patients were classified based on GCs receipt. Categorical variables were analyzed using Pearson's chi-squared (χ2) or Fisher's exact test and continuous variables were analyzed using non-parametric tests. A two-way repeated-measures analysis of variances (ANOVA) was utilized for assessing the changes in the peripheral environment over the postoperative course. Levels of lymphocyte subsets, lymphocyte phenotypes, lymphocyte functions, and inflammatory cytokines were compared between two groups using independent *T*-test or Mann-Whitney U test at each time point and one-way ANOVA or Kruskal Wallis test was used to compare between three groups. Propensity score matching (1:1) was used to achieve a balanced exposure group at baseline variables, age, sex, smoking status, tumor stage, and extent of resection were included in the regression model. Hosmer and Lemeshow test was used to check the goodness of fit of the regression model. Patients were matched based on the propensity score with a caliper width of 0.03. Two-sided *P*-values < 0.05 were considered statistically significant. Statistical analyses were performed using SPSS Version 23 for Windows (SPSS Inc, Chicago, USA) and Graph Pad Prism Software (Graph Pad Software, San Diego, USA).

## Results

### Clinicopathologic Characteristics and Administration of GCs

During the study period, 105 patients met the inclusion criteria, the majority of whom were female (62, 59%) with a median age of 64 years (interquartile range (IQR), 59–67). Clinicopathologic characteristics were generally well-balanced between those who did or did not receive GCs in terms of sex, age, smoking history, histology, tumor stage, EGFR mutation status, and extent of resection. Of these 105 patients, 67 (64%) received 109 times of GCs and 38 (36%) did not receive GCs. The mean total dose of GCs was 8.4 mg (IQR, 5–10) of intravenous dexamethasone equivalents. The indications for GCs were prevention of postoperative nausea and vomiting (62%), dyspnea or other respiratory symptoms (31%), and allergies (7%) (Table 1).

**Table 1:** Baseline characteristics of patients.

**Characteristics**	**Patients without GCs** **(*n* = 38)**	**Patients with GCs** **(*n* = 67)**	***P***
Sex			0.067
Male	20 (53)	23 (34)	
Female	18 (47)	44 (66)	
Median age, years	57 (52–65)	61 (52–65)	0.569
Smoking status			0.819
Never-smoker	27 (71)	50 (75)	
Ever-smoker	11 (29)	17 (25)	
Histology			0.979
Adenocarcinoma	35 (92)	62 (93)	
Squamous cell carcinoma	2 (5)	3 (4)	
Pleomorphic/Mixed histology	1 (3)	2 (3)	
Tumor stage			0.830
I	30 (79)	55 (82)	
II	6 (16)	10 (15)	
III	2 (5)	2 (3)	
EGFR mutation status			0.605
EGFR mutant	18 (48)	28 (42)	
EGFR wild type	10 (26)	15 (22)	
EGFR unknown	10 (26)	24 (36)	
Extent of resection			0.404
Sublobectomy	9 (24)	21 (31)	
Lobectomy	29 (76)	46 (69)	
Indications of GCs			NA
Prevention of postoperative nausea and vomiting		67 (62)	
Dyspnea or other respiratory symptoms		34 (31)	
Allergies		8 (7)	
Drug regimen			NA
A single dose of GCs		35 (52)	
Multiple doses of GCs		32 (48)	
Mean total dose of GCs (dexamethasone equivalents), mg		8.4 (5–10)	NA

*Data are presented as numbers (%), mean or median (interquartile range)*.

*GCs, glucocorticoids; EGFR, epidermal growth factor receptor; NA, not available*.

### Postoperative Complications

There were no 30-day mortalities and reoperations. There were no differences in the incidence of postoperative events between the GCs group and the no GCs group. The total chest tube output and chest output the first three days after surgery in the GCs group were slightly less than that in the no GCs group, although not statistically significant (Table 2).

**Table 2:** Postoperative complications.

**Complications**	**Patients without GCs** **(*n* = 38)**	**Patients with GCs** **(*n* = 67)**	***P***
Cardiovascular	1 (3)	2 (3)	1.000
Duration of chest tube drainage	5.9 (4.8–6.0)	6.2 (5.0–7.0)	0.588
Air leak >5 days	1 (3)	2 (3)	1.000
Postoperative pleural effusion requiring drainage	2 (5)	5 (8)	1.000
Total chest tube output, ml	1385 (749–1,439)	1176 (755–1,305)	0.259
Pneumothorax requiring drainage	1 (3)	2 (3)	1.000
Atelectasis	2 (5)	4 (6)	1.000
Pneumonia	2 (5)	5 (8)	1.000
Wound infection	1 (3)	3 (5)	1.000
Urinary tract infection	0 (0)	2 (3)	0.534
Other infections	0 (0)	1 (2)	1.000
Readmission to ICU	1 (3)	2 (3)	1.000

*Data are presented as numbers (%) or mean (interquartile range)*.

*GCs, glucocorticoids; ICU, intensive care unit*.

### Dynamic Changes of the Peripheral Environment After Surgery

**Figure 1:**
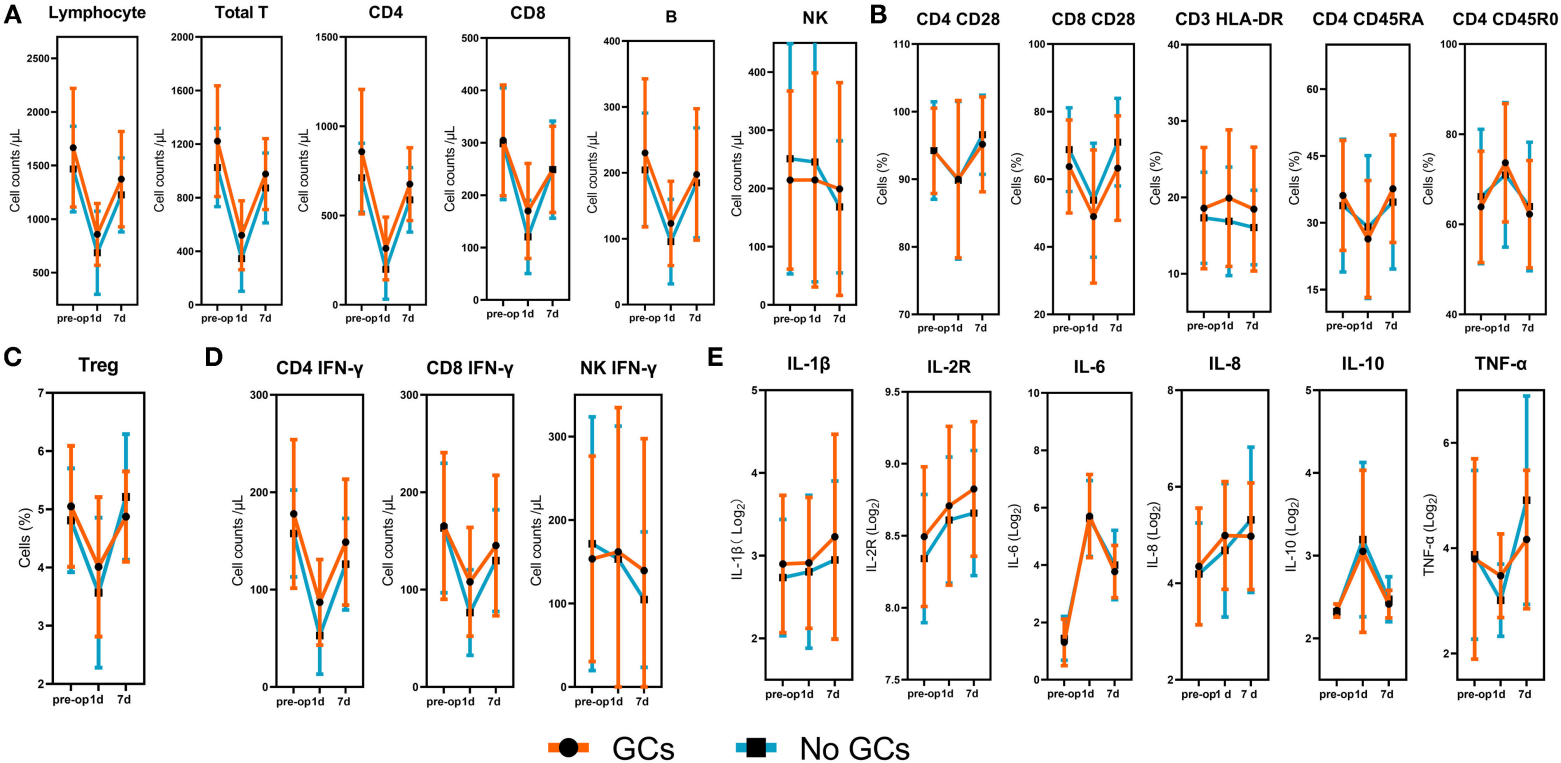
Dynamic changes of lymphocyte subsets, lymphocyte phenotypes, lymphocyte functions, and inflammatory factors. **(A)** The absolute numbers of total lymphocytes, T cells, B cells, NK cells, CD4^+^and CD8^+^T cells. **(B)** The expression of CD28 on CD4^+^ and CD8^+^ T cells, the expression of HLA-DR on CD3^+^ T cells, the expression of CD45RO and CD45RA on CD4+ T cells. **(C)** The percentages of Treg cells. **(D)** The absolute numbers of IFN-γ producing CD4^+^ T cells, CD8^+^ T cells, and NK cells. **(E)** Logarithms of cytokines based on 2. The *p*-values for the time trend were >0.05 for all immune cells and inflammatory cytokines.

Regarding the collection of blood samples, 105 (100%) patients had preoperative levels available, 88 (84%) had samples taken at 1 day postoperatively, and 85 (81%) had samples taken at 7 days postoperatively. When we assessed the changes of immune cells and inflammatory factors over the postoperative course, we found that all peripheral immune components included in this study significantly altered in response to surgery (Figure 1). The absolute numbers of total lymphocytes, T cells, B cells, CD4^+^ and CD8^+^ T cells significantly decreased at 1 day postoperatively. The recovery was detected at 7 days post-operatively, although still significantly lower than preoperative levels (Figure 1A). The levels of co-stimulatory molecule CD28 expressed by CD4^+^ and CD8^+^T cells, the expression of CD45RA on CD4^+^ T cells (Figure 1B), the percentage of Treg cells (Figure 1C), and IFN-γ producing CD4^+^ and CD8^+^T cells (Figure 1D) showed similar patterns, although varied in magnitude. The absolute numbers of NK cells and IFN-γ producing NK cells showed a gradual decrease over the assessed time.

Inflammatory factors were also measured at three time points to determine the dynamic changes of inflammation following surgery. A continuous increase was observed for IL- 1β, IL-2R, IL-8 within 7 days after lung resection. Levels of IL-6 and IL-10 were significantly increased at 1 day after surgery, followed by a reduction at 7 days postoperatively. Pro-inflammatory cytokine, TNF-α, showed no significant change at 1 day postoperatively but significantly increased at 7 days postoperatively (Figure 1E).

### Effect of Perioperative Use of GCs on Peripheral Environment

Assessment of each lymphocyte subset, lymphocyte phenotype, and lymphocyte function at preoperatively and 7 days postoperatively demonstrated similar values between groups.

**Figure 2:**
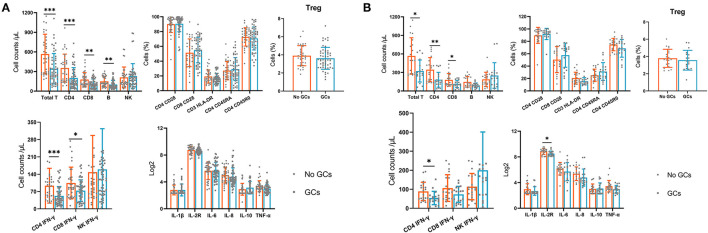
The lymphocyte subsets, lymphocyte phenotypes, lymphocyte functions and inflammatory cytokines in the GCs group and no GCs group at 1 day postoperatively. **(A)** Before propensity score matching. **(B)** After propensity score matching. *p < 0.05, **p < 0.01, ***p < 0.001.

Next, we compared the peripheral immune components at 1 day postoperatively between groups. We found that the absolute numbers of total T cells, CD4^+^ T cells, CD8^+^T cells, B cells, and IFN-γ producing CD4^+^ and CD8^+^T cells were significantly lower in the GCs group compared with the no GCs group. Analysis of lymphocyte phenotypes, inflammatory cytokines at 1 day postoperatively demonstrated similar values between groups (Figure 2A).

Furthermore, we used the PSM (1:1) and 22 pairs of patients were successfully matched (Table 3) then we compared the immune components again. Levels of immune cells and inflammatory cytokines were similar between the two groups preoperatively and at 7 days postoperatively. At 1 day postoperatively, assessment of immune cells and cytokines showed similar results to that before PSM (Figure 2B).

**Table 3:** Baseline characteristics after propensity-matching adjustment of confounding covariates.

**Characteristics**	**Patients without GCs** **(*n* = 22)**	**Patients with GCs** **(*n* = 22)**	***P***
Sex			0.728
Male	5 (23)	6 (27)	
Female	17 (77)	16 (73)	
Median age, years	63 (58–66)	64 (54–67)	0.849
Smoking status			0.761
Never-smoker	12 (55)	13 (59)	
Ever-smoker	10 (45)	9 (41)	
Histology			0.513
Adenocarcinoma	20 (91)	20 (91)	
Squamous cell carcinoma	1 (5)	2 (9)	
Pleomorphic/Mixed histology	1 (5)	0 (0)	
Tumor stage			0.293
I	20 (91)	19 (86)	
II	1 (5)	3 (14)	
III	1 (5)	0 (0)	
EGFR mutation status			0.470
EGFR mutant	5 (23)	8 (36)	
EGFR wild type	7 (32)	4 (18)	
EGFR unknown	10 (46)	10 (46)	
Extent of resection			0.680
Sublobectomy	3 (14)	4 (18)	
Lobectomy	19 (86)	18 (82)	

*Data are presented as numbers (%), median (interquartile range)*.

*GCs, glucocorticoids; EGFR, epidermal growth factor receptor*.

### Effect of Multiple Doses of GCs on T Cells and B Cells

**Figure 3:**
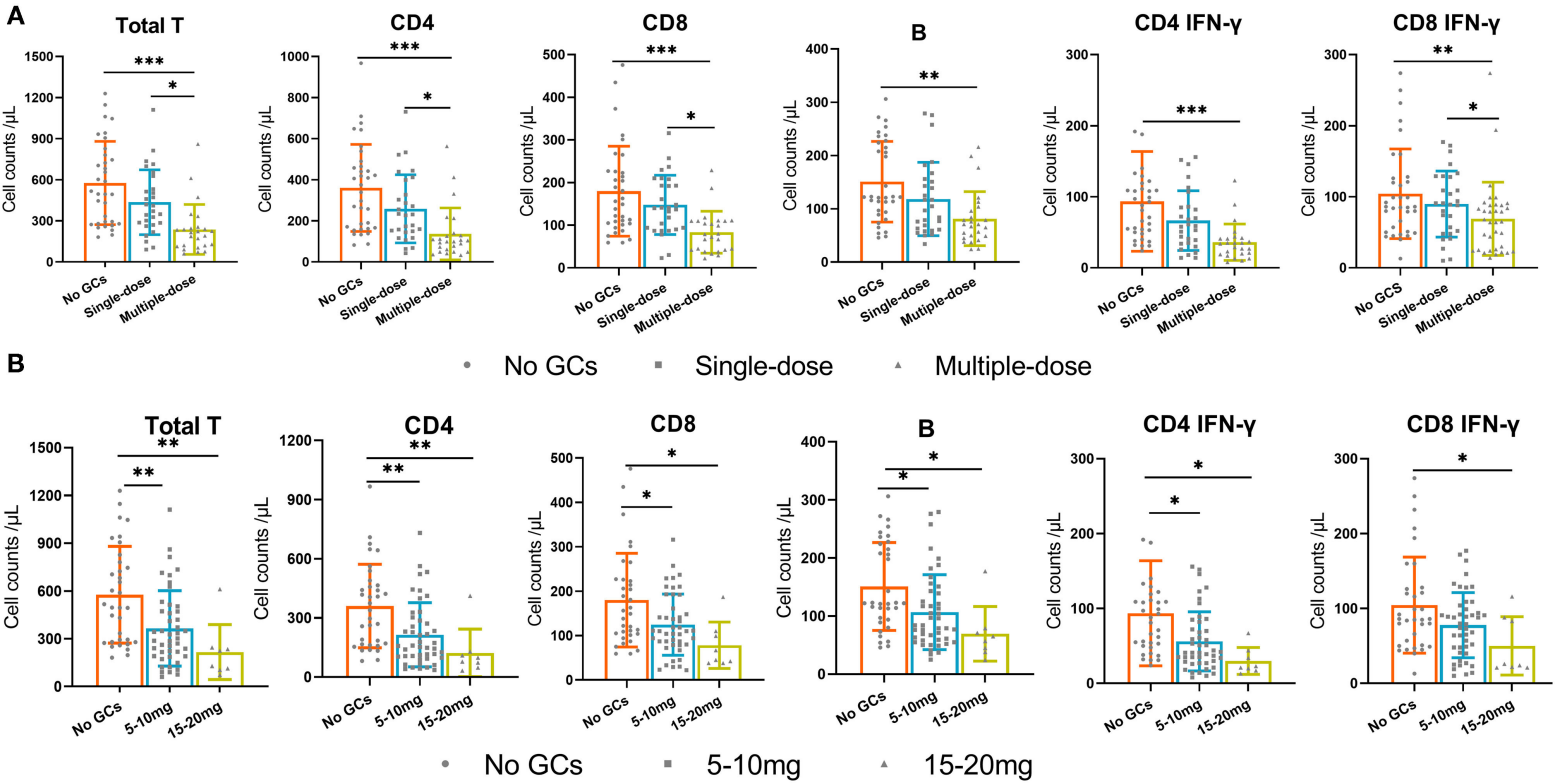
The absolute numbers of total T cells, CD4^+^T cells, CD8^+^T cells, B cells, and IFN-γ producing CD4^+^ T cells and CD8^+^ T cells in three groups. **(A)** the no GCs group, single-dose group, and multiple-dose group. **(B)** the no GCs group, 5–10 mg group, and 15–20 mg group. *p < 0.05, **p < 0.01, ***p < 0.001.

In the GCs group, 35 (52%) patients received an intraoperative single dose of GCs and 32 (48%) patients received multiple doses of GCs. We found that multiple doses of GCs were significantly associated with the decreased absolute number of total T cells, CD4^+^ T cells, CD8^+^T cells, B cells, and IFN-γ producing CD4^+^ and CD8^+^T cells while a single dose was not (Figure 3A). Of note, the dosage of GCs in all patients who received an intraoperative single dose GCs is the same 5 mg dexamethasone.

### Effect of High Doses of GCs on T Cells and B Cells

Since the recommended dosage of dexamethasone used in surgery ranges between 4 and 10 mg (9), we then divided the GCs group into 5–10 mg group and 15–20 mg group. We found that the use of 5–10 mg of GCs was significantly associated with the decreased absolute number of total T cells, CD4^+^ T cells, CD8^+^T cells, B cells, and IFN-γ producing CD4^+^ T cells. The absolute numbers of immune cells in the 15–20 mg group were lower than that in the 5–10 mg group, although not statistically significant (Figure 3B).

### Effect of Multiple Doses of GCs on Lymphocyte Phenotypes and Inflammatory Cytokines

**Figure 4:**
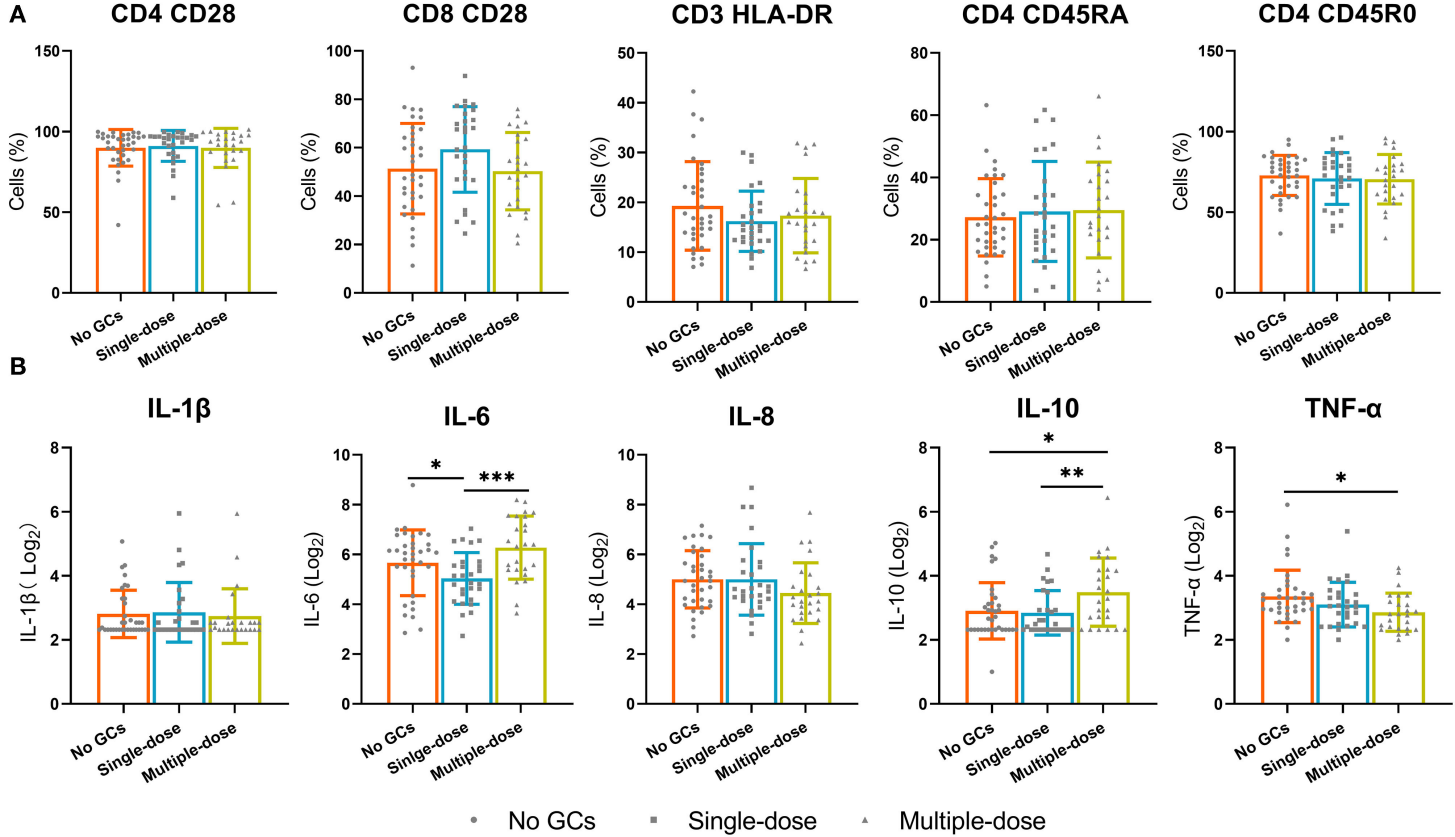
The lymphocyte phenotypes and known inflammatory cytokines in the no GCs group, single-dose group, and multiple-dose group. **(A)** The expression of CD28 on CD4^+^ and CD8^+^ T cells, the expression of HLA-DR on CD3^+^ T cells, the expression of CD45R0 and CD45RA on CD4+ T cells. **(B)** Inflammatory factors. *p < 0.05, **p < 0.01, ***p < 0.001.

Given the effect of multiple doses of GCs on T cells and B cells, we examined the effect of multiple doses of GCs on lymphocyte phenotypes and known inflammatory cytokines. Lymphocyte phenotypes were still not influenced by the use of a single dose or multiple doses of GCs (Figure 4A). Increased levels of IL-1β and IL-10 were observed in the multiple-dose group and lower levels of TNF-α were observed in the multiple-dose group (Figure 4B).

## Discussion

We report that the perioperative administration of GCs is associated with aggravated immunosuppression in patients with NSCLC. Furthermore, we demonstrated that multiple doses or high doses (15–20 mg dexamethasone equivalents) of GCs that were all given within 24 h were strongly associated with decreased absolute numbers of T cells, CD4^+^ T cells, CD8^+^T cells, and B cells and impaired T cells functions while a single intraoperative low dose (5 mg dexamethasone equivalents) was not. Inflammatory cytokines were also more affected by multiple doses of GCs. Infectious outcomes appear to be not influenced by the use of GCs.

Lymphocyte subsets are central components of the immune network and play a critical role in the elimination of pathogens and circulating cancer cells. However, major surgery for cancer is known to induce lymphopenia and suppress lymphocyte function (18). Similar to previous studies (19, 20), we demonstrated a significant decrease in peripheral lymphocyte subpopulations at 1 day postoperatively in all patients. IFN-γ production has been demonstrated to be well-correlated with various activities of lymphocytes and can be utilized as a symbol of lymphocyte function (17). In the present study, we assessed the changes of IFN-γ producing lymphocytes over surgery course and observed decreased IFN-γ producing lymphocytes. These results suggest that lymphocyte subsets count and lymphocyte functions are suppressed following surgery in patients with NSCLC.

Other studies have yielded similar findings to the present report with respect to inflammatory cytokines. In a prospective randomized trial that evaluated 41 patients undergoing VATS or thoracotomy, Craig et al. demonstrated increased CRP and IL-6 in both groups after surgery, and the response of the IL-6 and CRP to surgery both showed a lower peak concentration in the minimally invasive surgery group compared with the conventional open surgery group (21). Yim et al. also reported that the levels of IL-6, IL-8, and IL-10 were elevated postoperatively (22).

These changes in lymphocyte subpopulations and inflammatory cytokines during the perioperative period are believed to contribute to immunosuppression in the early postoperative period. The impact of postoperative immunosuppression on infectious complications and cancer occurrence has increasingly become an important and potentially modifiable factor (23). Minimally invasive approaches are considered to be an important factor that can decrease inflammation in response to surgery compared with open surgery (22). However, the impact of the surgical approach on long-term oncologic results remains controversial (24–26), and perhaps other perioperative factors including administration of specific drugs may be crucial in determining disease-free survival.

GCs are hormones that play a critical physiologic role in immune system homeostasis and are commonly used in surgical patients to treat a variety of indications for their anti-inflammatory properties. GCs are also known for their immunosuppressive properties, and exogenous GCs have been demonstrated to downregulate inflammatory cytokines and induce the apoptosis of lymphocytes (27). These effects can offer substantial clinical benefit in the treatment of many diseases, such as COVID-19 (28), but may have unintended consequences in oncological patients undergoing surgery.

Few studies have evaluated the effect of perioperative use of GCs on the peripheral environment following surgery. In a prospective, double-blinded randomized controlled trial, Corcoran et al. randomized 32 patients to control group or dexamethasone group, and measured peripheral immune cells and molecules preoperatively and at 24h, 48h, and 6 weeks post-operatively (29). Lymphocyte counts, T cell counts, and B cell counts showed a decrease in both groups at 24 h and resolved at 48 h, which were similar to our findings. However, they reported that the decreases in lymphocyte counts, T cell counts, and B cell counts were greater in the control group than in the dexamethasone group, which was contrary to our findings. The discrepancies may arise from different drug regimens, the previous study evaluated the effect of a single lower dose (4 mg) of dexamethasone on the peripheral environment. In the present study, we found that high doses or multiple doses of GCs were strongly associated with decreased count of immune cells while a single low dose was not. Furthermore, GCs have been demonstrated to have both immunostimulatory and immunosuppressive effects (30), which may account for part of the discrepancy between the two studies.

Changes in immune cell absolute numbers do not necessarily reflect changes in immune function. We, therefore, analyzed the effects of GCs on lymphocyte functions. The results showed that the IFN-γ producing CD4^+^ and CD8^+^ T cells were significantly lower in the GCs group than in the no GCs group. Further assessment revealed that multiple doses of GCs were strongly associated with impaired T cells functions while an intraoperative single dose of GCs was not. These results suggest that the use of multiple doses of GCs result in both decreased numbers of immune cells and suppression of T cells functions.

Excessive production of many inflammatory factors is normally counteracted by GCs in many diseases and healthy volunteers (31, 32). In the initial examination of inflammatory cytokines in the present study, inflammatory cytokines appeared to be not influenced by the treatment of GCs. However, after dividing the GCs group into the single-dose group and multiple-dose group, levels of IL-6 and IL-10 were higher in the multiple-dose group. There is no mechanistic explanation for this finding, further investigation is needed.

Important outcomes related to perioperative factors including administration of GCs are the focus of much attention, particularly infectious complications and cancer recurrence. Suppression of host defense mechanism plays a critical role in both outcomes. Similar to previous studies (33, 34), our investigation suggests that perioperative administration of GCs does not result in an increased incidence of infectious complications following surgery. It appears that perioperative use of GCs also has few adverse effects on cancer-related outcomes (14, 15). However, most of these studies investigated the effect of an intraoperative single low dose of GCs on infectious complications and long-term outcomes. In the present study, we found that 32 (48%) patients received multiple doses of GCs within 24 h among patients who received GCs and we already excluded a proportion of patients who received GCs between the 1 day and 7 days postoperatively considering the comparability between the two groups. The Synergistic effects of GCs use and surgery in immunosuppression may allow disseminated tumor cells to survive in the circulation and increase their chances of developing into metastases. Furthermore, although the effect of GCs on immune cells is transient, multiple doses of GCs over the entire postoperative course could prolong the aggravated immunosuppression and may affect long-term cancer-related outcomes.

There are several limitations to this study. First, the study is limited by its retrospective nature and small sample size. Second, a few patients did not complete blood collection at 1 day and 7 days postoperatively, which may limit the accuracy. Third, our study included a high proportion of patients with early-stage lung cancer, the extent to which our findings can be generalized to locally advanced lung cancer remains unknown.

In summary, perioperative multiple doses or relatively high doses (15–20 mg dexamethasone equivalents) of GCs that are all given within 24 h are associated with decreased absolute numbers of T cells, CD4^+^ T cells, CD8^+^T cells, and B cells and impaired T cells functions while a single intraoperative low dose (5 mg dexamethasone equivalents) is not. Our findings suggest that multiple doses or high doses of GCs within a short time should be avoided.

## Data Availability Statement

The original contributions presented in the study are included in the article/supplementary material, further inquiries can be directed to the corresponding author/s.

## Ethics Statement

The studies involving human participants were reviewed and approved by Medical Ethics Committee of Tongji Hospital, Tongji Medical College, Huazhong University of Science and Technology (TJ-IRB20210837). The patients/participants provided their written informed consent to participate in this study.

## Author Contributions

XF contributed to the study design and revised the article. LY and YC collected the data and analyzed and interpreted the data. XF and YC helped to draft the article. LY wrote the article. All authors read and approved the final manuscript.

## Conflict of Interest

The authors declare that the research was conducted in the absence of any commercial or financial relationships that could be construed as a potential conflict of interest.

## Publisher's Note

All claims expressed in this article are solely those of the authors and do not necessarily represent those of their affiliated organizations, or those of the publisher, the editors and the reviewers. Any product that may be evaluated in this article, or claim that may be made by its manufacturer, is not guaranteed or endorsed by the publisher.
